# Stochastic undersampling steepens auditory threshold/duration functions: implications for understanding auditory deafferentation and aging

**DOI:** 10.3389/fnagi.2015.00063

**Published:** 2015-05-15

**Authors:** Frédéric Marmel, Medardo A. Rodríguez-Mendoza, Enrique A. Lopez-Poveda

**Affiliations:** ^1^Audición Computacional y Psicoacústica, Instituto de Neurociencias de Castilla y León, Universidad de SalamancaSalamanca, Spain; ^2^Grupo de Audiología, Instituto de Investigación Biomédica de Salamanca, Universidad de SalamancaSalamanca, Spain; ^3^Facultad de Medicina, Departamento de Cirugía, Universidad de SalamancaSalamanca, Spain

**Keywords:** auditory deafferentation, auditory aging, auditory neuropathy, stochastic sampling, temporal processing, temporal integration

## Abstract

It has long been known that some listeners experience hearing difficulties out of proportion with their audiometric losses. Notably, some older adults as well as auditory neuropathy patients have temporal-processing and speech-in-noise intelligibility deficits not accountable for by elevated audiometric thresholds. The study of these hearing deficits has been revitalized by recent studies that show that auditory deafferentation comes with aging and can occur even in the absence of an audiometric loss. The present study builds on the stochastic undersampling principle proposed by Lopez-Poveda and Barrios (2013) to account for the perceptual effects of auditory deafferentation. Auditory threshold/duration functions were measured for broadband noises that were stochastically undersampled to various different degrees. Stimuli with and without undersampling were equated for overall energy in order to focus on the changes that undersampling elicited on the stimulus waveforms, and not on its effects on the overall stimulus energy. Stochastic undersampling impaired the detection of short sounds (<20 ms). The detection of long sounds (>50 ms) did not change or improved, depending on the degree of undersampling. The results for short sounds show that stochastic undersampling, and hence presumably deafferentation, can account for the steeper threshold/duration functions observed in auditory neuropathy patients and older adults with (near) normal audiometry. This suggests that deafferentation might be diagnosed using pure-tone audiometry with short tones. It further suggests that the auditory system of audiometrically normal older listeners might not be “slower than normal”, as is commonly thought, but simply less well afferented. Finally, the results for both short and long sounds support the probabilistic theories of detectability that challenge the idea that auditory threshold occurs by integration of sound energy over time.

## Introduction

Hearing impairment is routinely diagnosed on the basis of elevated audiometric thresholds, i.e., on the basis of an increase in the lowest sound levels at which listeners can detect pure tones of different frequencies. It has long been known, however, that listeners can experience hearing difficulties not reflected in their audiometric thresholds (Kopetzky, 1948; King, 1954; for reviews see Lopez-Poveda, 2014; Plack et al., 2014). These hearing impairments are sometimes referred to as “hidden” hearing losses (Schaette and McAlpine, 2011). They include hyperacusis and tinnitus (Schaette and McAlpine, 2011) as well as deficits in temporal processing and related abilities, such as sound localization, temporal resolution, and/or speech-in-noise perception (Starr et al., 1991; Kraus et al., 2000; Zeng et al., 2005; Zeng and Liu, 2006; Zhao and Stephens, 2007). Behaviorally, studies of patients diagnosed with auditory neuropathy have well documented the association between impaired speech-in-noise perception and impaired temporal processing (Starr et al., 1991; Kraus et al., 2000; Zeng et al., 2005; Zeng and Liu, 2006). Impaired speech-in-noise perception and impaired temporal processing are also a frequent concern for older listeners with (near) normal hearing thresholds (CHABA, 1988; Pichora-Fuller and MacDonald, 2008; Fitzgibbons and Gordon-Salant, 2010; Humes and Dubno, 2010). Physiologically, recent studies have reported that noise exposure causes a permanent loss of auditory nerve fibers even though audiometric thresholds recover rapidly to the normal range (Kujawa and Liberman, 2009; Lin et al., 2011; Furman et al., 2013). Kujawa and Liberman (2009) argued that this deafferentation should “decrease the robustness of stimulus coding in low signal-to-noise conditions, for example speech in noise, where spatial summation via convergence of activity from groups of neurons must be important in signal processing” (p. 14083). Deafferentation also occurs with aging (Makary et al., 2011; Sergeyenko et al., 2013), which suggests that deafferentation could contribute to the speech-in-noise and temporal processing deficits observed in older listeners with (near) normal audiometry. The present study investigates how deafferentation could deteriorate temporal processing, in particular the detection of brief sounds. The present study also contributes to our understanding of the mechanisms underlying sound detection.

Lopez-Poveda and Barrios (2013) proposed a signal-processing analogy based on the stochastic nature of action potentials to explain how deafferentation could result in poorer temporal processing and speech-in-noise perception. They noted that the stochastic nature of action potentials imposes a limit to information encoding in the auditory nerve. Action potentials being stochastic means that individual auditory nerve fibers (ANFs) do not perfectly sample the waveform of the mechanical cochlear response in the cochlear region innervated by the fiber. Instead, an ANF can be seen as providing an undersampled, incomplete, representation of the mechanical response waveform in question. In a normal auditory nerve, a high-quality waveform representation would be granted by the pooling of the spike trains from all the ANFs in the nerve. Such a pooling mechanism is reminiscent of the “volley theory” (Wever, 1949) and has been shown to be effective for the encoding of speech sounds (Stevens and Wickesberg, 1999, 2002). In a deafferented nerve, however, the reduced number of ANFs would be less able to compensate for the limited information encoded by individual fibers.

In addition, Lopez-Poveda and Barrios (2013) argued that the stochastic nature of action potentials implies that a reduction of the number of ANFs would specifically degrade the coding of low-intensity and high-frequency sound features. They reasoned that the stochastic nature of action potentials means that the quantity of stimulus information conveyed by an individual ANF depends (1) on its instantaneous probability of firing as a function of stimulus intensity; and (2) on the stimulus duration. As the probability of an individual ANF firing increases with increasing sound pressure (Sachs and Abbas, 1974; Heil et al., 2011), a given ANF would be more likely to convey high-intensity than low-intensity sound features. Also, as action potentials occur stochastically in time, the probability of an individual ANF firing at least once in response to a stimulus increases with increasing the stimulus duration (Heil et al., 2008), which means that an individual ANF would be more likely to fire in response to a long sustained stimulus than to a short transient stimulus of equal intensity. As a result, acoustic features involving long intervals (low frequency features) would be more likely to be represented in the spike train of an individual ANF than acoustic features involving short intervals (high frequency features). In case of deafferentation, the comparatively fewer surviving ANFs might be insufficient to compensate for the limited information encoding of low-intensity and high-frequency features by individual ANFs.

**Figure 1:**
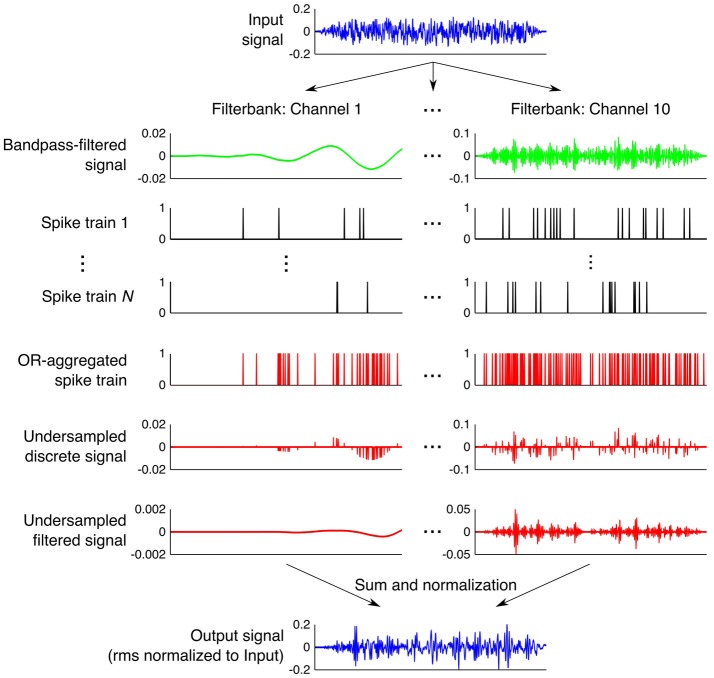
**Step-by-step illustration of the processing done by the stochastic undersampling vocoder**. See Section Stochastic Undersampling Vocoder for a description.

Lopez-Poveda and Barrios (2013) tested their theory experimentally with a vocoder (see Section Stochastic Undersampling Vocoder, and Figure 1) designed to generate *N* stochastically undersampled versions of the stimulus per frequency channel, where the parameter *N* is the number of stochastic samplers and would roughly simulate *N* auditory nerve fibers. Lopez-Poveda and Barrios (2013) measured pure tone detection thresholds and speech recognition in quiet and in noise in young normal-hearing listeners, for stimuli processed with either a large or a small number of simulated fibers. Undersampled and non-processed stimuli were equalized for root-mean-square (rms) amplitude to make sure that performance was independent of differences in overall stimulus intensity. Instead, differences in performance would reflect changes in the distribution of stimulus energy along time, i.e., changes in the stimulus waveforms. Reducing the number of simulated fibers impaired speech recognition in noise but not in quiet, consistent with older listeners’ impaired speech-in-noise perception (CHABA, 1988; Humes and Dubno, 2010). Pure-tone detection was slightly impaired both in noise and in quiet but detection thresholds were still within the normal range, which is consistent with the threshold recovery observed in noise-induced deafferentation studies (Kujawa and Liberman, 2009; Lin et al., 2011). These two results suggested that stochastic undersampling is a reasonable analogy to explain how auditory deafferentation would cause speech-in-noise difficulties in listeners with (near) normal audiometric thresholds. The present study uses the stochastic undersampling analogy of Lopez-Poveda and Barrios (2013) to investigate how deafferentation could impair specific aspects of temporal processing.

We focus on threshold/duration functions, which are often referred to as “temporal integration” functions and describe the phenomenon of higher detection thresholds for shorter than for longer sound durations (Hughes, 1946; Garner and Miller, 1947). Auditory neuropathy patients have abnormally elevated detection thresholds for shorter durations (below approximately 30 ms) resulting in steeper threshold/duration functions than control listeners (Starr et al., 1991; Zeng et al., 2005). Even though “auditory neuropathy” is not always caused by alterations to the auditory nerve (Starr, 2009), deafferentation could be one possible cause of the steeper threshold/duration functions observed in these patients, given that the stochastic undersampling analogy of deafferentation predicts that short sounds are less likely to be represented in the response of ANFs than are long sounds. Poorer detection of short pure tones (15 ms) has also been reported to be a predictor of speech-in-noise perception in a group of listeners covering a wide range of ages (89 listeners, 21–82 years) whose thresholds were within the normal range for their age and for whom detection thresholds for longer (50 ms) tones did not correlate with age (Fostick and Babkoff, 2013; Fostick et al., 2013). The present study used the vocoder implementation of the stochastic undersampling principle (Lopez-Poveda and Barrios, 2013) to measure threshold/duration functions as a function of the degree of stochastic undersampling. As will be shown, reducing the number of stochastic samplers resulted in steeper threshold/duration functions. Therefore, stochastic undersampling, and so presumably deafferentation, could explain how deafferented listeners have trouble detecting short transient sounds.

We note that although threshold/duration functions are often referred to as “temporal integration” functions, the seminal explanation for absolute threshold that assumes that the auditory system integrates sound intensity over time (Green et al., 1957; Plomp and Bouman, 1959) has been challenged several times. Alternative mechanisms have been proposed: (1) the quantity integrated over time could be sound pressure rather than sound intensity (Heil and Neubauer, 2003); (2) there could be no long-term integration but instead a series of short “multiple looks”, each providing independent information to be stored in memory and combined intelligently across looks (Viemeister and Wakefield, 1991); and (3) there could be no integration at all but instead a probability accumulation over time that would require no memory, with thresholds corresponding to the occurrence of a criterion number of stochastic detection events (Heil and Neubauer, 2003; Heil et al., 2013b) or even to one single detection event (Meddis and Lecluyse, 2011). In the present study, undersampled and non-processed stimuli were equated for rms amplitude (as in Lopez-Poveda and Barrios, 2013); hence stimuli of the same duration but processed with different degrees of stochastic undersampling will have the same energy and so any difference in their detectability will not be consistent with mechanisms based on long-term integration of intensity. Instead, the stochastic undersampling principle is reminiscent of the probabilistic approaches of sound detectability (Meddis and Lecluyse, 2011; Heil et al., 2013a,b) as they share the principle of enhanced stimulus representations for larger amplitudes and longer stimuli. A reduction in the number of stochastic samplers, which we use to simulate deafferentation, could be thought of as leading to a less efficient probability accumulation.

## Materials and Methods

### Participants

Nine participants (5 females) were tested. Their ages ranged from 24 to 33 years, with a mean of 27 years. All of them had audiometric thresholds less than 20 dB HL at octave frequencies spanning 250–8000 Hz (American National Standards Institute, 2004) and none reported any history of hearing impairment. All participants were tested in their right ear. Subjects were volunteers and were not paid for their service.

### Stochastic Undersampling Vocoder

Figure 1 illustrates stochastic undersampling with *N* = 10 samplers, for a 20-ms broadband noise with an rms amplitude of 0.495 (which corresponds to a presentation level of 100 dB SPL with our apparatus). The noise was filtered through a bank of ten fourth-order Butterworth filters (only two are shown in Figure 1) with cut-off frequencies logarithmically spaced between 100 Hz and 10 kHz to roughly mimic frequency decomposition within the cochlea. For each filter output, multiple (*N*) “spike” trains were stochastically generated to roughly mimic *N* different possible encodings of the signal by *N* different “afferent fibers” innervating a given cochlear region. Each “spike” train was obtained by sample-wise comparisons of the absolute amplitude of the filtered signal (all “digital” amplitudes between 0 and 1) with an equal-length array of random numbers uniformly distributed between 0 and 1. A unity-amplitude “spike” was generated whenever the signal’s absolute amplitude exceeded the corresponding random number. Thus, signals of higher intensities were more likely to generate “spikes” than signals of lower intensities. The resulting *N* “spike” trains per frequency band were aggregated into a single “spike” train using a sample-wise logical OR function; that is, unity amplitude “spikes” occurred in the aggregated response whenever a “spike” occurred in *any* of the *N* available “spike” trains. Thus, the larger the number of stochastic samplers (*N*), the more likely the aggregated response was to contain “spikes”. An acoustic version of the aggregated “spike” train was then obtained by sample-wise multiplication of the train in question with the output of the filter in each band. The reconstructed signal from each frequency band was then filtered through its corresponding Butterworth filter to filter out distortion or energy splatter. Finally, the ten resulting signals (one per band) were sample-wise added to obtain a vocoded stimulus, whose rms amplitude was normalized to the rms amplitude of the original stimulus so that stochastic undersampling would only affect the temporal distribution of energy and not the overall stimulus energy. For low-intensity signals and/or when using a small number *N* of stochastic samplers, it could happen that stochastic undersampling did not preserve any of the samples in the original stimulus (no “spikes” were generated). In those cases, rms normalization was not applied and the processed stimuli were left blank (a condition akin to having no stimulus).

### Stimuli

All stimuli were broadband (20–10000 Hz) noises and had 2.5-ms cosine squared onset and offset ramps. Detection thresholds were measured as a function of stimulus duration, for stimulus duration (including the 2.5-ms ramps) of 5, 10, 20, 50, 100, 200, and 500 ms. Three different degrees of deafferentation were simulated by vocoding the stimuli with either 300 stochastic samplers per frequency channel, 1000 stochastic samplers per frequency channel, or by vocoding the stimuli without undersampling. As the undersampling of long stimuli was too computationally expensive to be made in real time, all stimuli were pre-generated so that the experimental software only had to load them from the computer hard drive. All stimuli were pre-generated for all sound levels between −10 and 80 dB SPL in 2-dB steps. For each level, three stimuli were pre-generated to avoid having exactly the same stimuli presented on different trials and—for each trial—the experimental software picked one of the three randomly. Some of the lower-intensity stimuli were left blank by the stochastic undersampling, mostly for short durations. As only three stimuli were generated per condition, the proportions of blank stimuli presented in the present study may have been different than what they would have been if the stimuli had been generated in real time. The implications of blank stimuli will be discussed in Section Implications for Mechanisms of Sound Detectability.

### Apparatus

All stimuli were generated digitally using custom Matlab software (The Mathworks, Natick, Massachusetts, USA). Stimuli were digital-to-analog converted using an RME Fireface 400 sound card at a sampling rate of 44100 Hz and a resolution of 24 bits, and presented monaurally through circumaural Sennheiser HD580 headphones. Subjects sat in a double-wall sound booth during testing. Stimulus intensity (in dB SPL) was specified in reference to the acoustic sound level of a 1-kHz digital sinusoidal wave with maximal digital amplitude (i.e., peak amplitudes equal to −1/+1). This calibration value was measured by placing the headphones on a KEMAR equipped with a Zwislocki coupler (Knowles DB-100) connected to a sound level meter (B&K 2238).

### Procedure

The experimental procedure was controlled via custom Matlab software. Detection thresholds were estimated in a three-interval-three-alternative forced choice task (3I3AFC) using a two-down one-up adaptive procedure, which tracks the 70.7% point on the psychometric function (Levitt, 1971). One interval (randomly chosen) contained the stimulus while the two other intervals were silent. Lights flashing on a computer screen marked the three observation intervals. The lights had the same duration as the stimulus and were separated by 500 ms. Listeners were asked to identify which interval contained the stimulus by pressing the corresponding key on a computer keyboard. Visual feedback indicated whether their response was right or wrong. The stimulus level was initially set to 60 dB SPL and varied adaptively in 6-dB steps for the first three reversals and in 2-dB steps for the next nine reversals. The mean and the standard deviation of the stimulus levels on the last eight reversals were calculated. If the standard deviation was less than 6 dB, the mean was taken as an estimate of the detection threshold. Three such estimates were obtained for each experimental condition and their mean was taken as the final threshold.

Experimental procedures were approved by the Ethics Review Board of the University of Salamanca.

### Statistical Analysis

Detection thresholds were compared with a repeated measures analysis of variance (ANOVA), using the degree of undersampling (without undersampling/300 samplers/1000 samplers) and the duration of the stimuli (5/10/20/50/100/200/500 ms) as within-subjects factors. Statistical results are reported with the Greenhouse-Geisser correction as the sphericity assumption was violated for duration.

Thresholds obtained for a given stimulus duration were compared across deafferentation conditions with *post hoc* two-tailed paired *t*-tests. The slopes of the threshold/duration functions were estimated for each participant and undersampling condition by fitting straight lines to the data (equations of the form: *threshold* = *a* + *b* × ln[*duration*]). For convenience, the estimated slopes will be reported as the decrease in threshold (in dB) per doubling duration (given by *b* × ln[2]). The mean slopes for each undersampling condition were compared using two-tailed paired *t*-tests.

## Results

**Figure 2:**
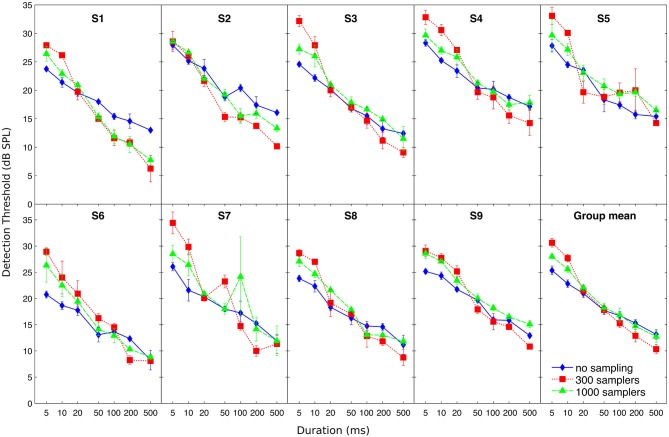
**Individual and mean detection thresholds as a function of stimulus duration**. Different symbols illustrate detection thresholds for different undersampling conditions (no sampling, 300 samplers, and 1000 samplers), as indicated by the inset in the bottom-right panel. Error bars show ±1 standard error of the mean calculated over the three threshold estimates (for individual thresholds) or over the final individual threshold estimates (for the group mean).

Individual and mean detection thresholds are plotted in Figure 2, which shows the data as analyzed statistically. To facilitate the comparison with figures from previous related studies (Florentine et al., 1988; Zeng et al., 1999, 2005), Figure 3 re-plots mean detection thresholds relative to the mean thresholds obtained for the 500-ms stimuli. All listeners showed thresholds that decreased with increasing duration in all conditions. Undersampling, however, affected thresholds differently for durations shorter and longer than about 20–50 ms. For durations <20 ms, eight out of nine listeners (listener S2 being the exception) had lower thresholds without undersampling than when using 300 samplers, and thresholds obtained with 1000 samplers were intermediate. Results for durations longer than 20 ms were less consistent across listeners, but overall the differences between undersampling conditions decreased and sometimes reversed as most listeners had thresholds without undersampling that were comparable to or higher than at least one of the two undersampling conditions.

**Figure 3:**
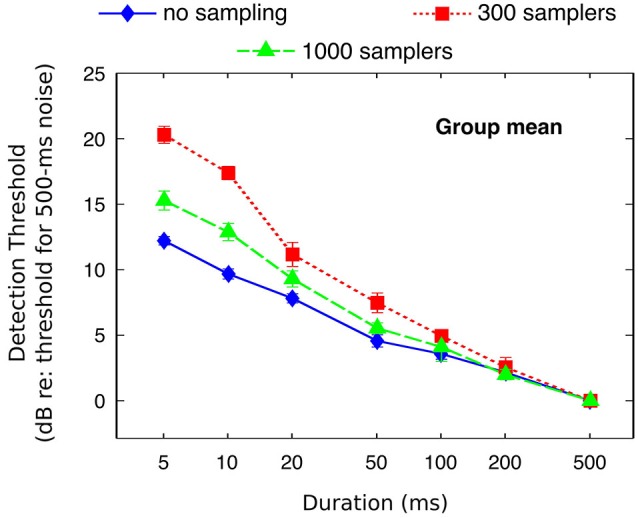
**Mean detection thresholds (from Figure 2) referenced to the thresholds for the 500-ms stimuli**.

The ANOVA revealed a main effect of duration (*F*_(6,48)_ = 366; *p* < 0.001) but not of undersampling (*F*_(2,16)_ = 2.27; *p* = 0.160). Instead, duration and undersampling interacted. The effect of duration was larger with undersampling than in the control condition, and larger when using 300 samplers than with 1000 samplers (*F*_(12,96)_ = 12.88; *p* < 0.001). Thresholds for short sounds were higher with undersampling than without it, contrary to thresholds for long sounds that were lower with undersampling than without it. For short sounds of 5 and 10 ms, *post hoc* paired *t*-tests showed that thresholds were significantly lower without undersampling than with 1000 samplers (5 ms: *t*_(8)_ = −5.63; *p* < 0.001; 10 ms: *t*_(8)_ = −7.11; *p* < 0.001). They also revealed that thresholds were significantly lower with 1000 than with 300 samplers (5 ms: *t*_(8)_ = −4.01; *p* < 0.005; 10 ms: *t*_(8)_ = −4.53; *p* < 0.005). For long sounds of 200 and 500 ms, *post hoc* paired *t*-tests showed that thresholds were significantly higher without undersampling than with 300 samplers (200 ms: *t*_(8)_ = 2.63; *p* < 0.05; 500 ms: *t*_(8)_ = 3.74; *p* < 0.01). Thresholds were also higher with 1000 than with 300 samplers (200 ms: *t*_(8)_ = 3.56; *p* < 0.01; 500 ms: *t*_(8)_ = 5.70; *p* < 0.001). However, thresholds obtained with 1000 samplers were not different from thresholds obtained without undersampling (200 ms: *t*_(8)_ = 0.75; *p* = 0.48; 500 ms: *t*_(8)_ = 0.55; *p* = 0.60).

**Figure 4:**
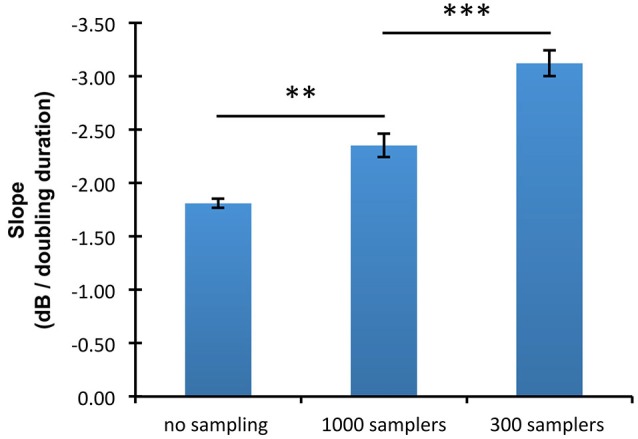
**Mean slopes of the threshold/duration functions in the three undersampling conditions (no sampling, 300 samplers, and 1000 samplers)**. Error bars show ±1 standard error of the mean calculated over the slopes estimated for each participant. Horizontal lines indicate the statistical comparisons made: two asterisks indicate a level of significance of *p* < 0.01, and three asterisks a level of significance of *p* < 0.001.

The slopes of the threshold/duration functions (Figure 4) indicated that thresholds in the absence of undersampling decreased by 1.81 dB for every doubling of duration whereas thresholds obtained with 300 and 1000 samplers decreased by respectively 3.12 and 2.35 dB for every doubling of duration. Hence, threshold/duration functions in the 1000 samplers and in the 300 samplers condition had slopes respectively 1.30 times and 1.73 times steeper than in the no undersampling condition. *Post hoc* paired *t*-tests confirmed shallower slopes in the absence of undersampling than when 1000 samplers were used (*t*_(8)_ = 4.07; *p* < 0.01), as well as shallower slopes when 1000 rather than 300 samplers were used (*t*_(8)_ = 6.96; *p* < 0.001).

## Discussion

### Stochastic Undersampling Impairs the Detection of Short Sounds, as Observed for Auditory Neuropathy Patients and Older Adults

We have shown that reducing the number of stochastic samplers leads to steeper threshold/duration functions with increased thresholds for the two shortest durations tested (5 and 10 ms). This result is consistent with the elevation of detection thresholds for short sounds observed in patients diagnosed with auditory neuropathy (Starr et al., 1991; Zeng et al., 2005) and in older listeners with (near) normal audiometric thresholds (Fostick et al., 2013). Zeng et al. (2005) measured threshold/duration functions for broadband noise in a group of normal-hearing listeners and in a group of auditory neuropathy patients and found that the latter had elevated thresholds for stimuli with durations of 5 and 10 ms. The slopes of the threshold/duration functions were 1.3 times steeper for the patients than for the normal-hearing listeners (−3.9 vs. −3.0 dB per doubling duration); the same ratio observed here between slopes for the 1000-samplers and the no-undersampling conditions. The present results are also consistent with elevated detection thresholds observed for short 1-kHz tones in older listeners with audiometric thresholds in the normal range for their age (Fostick and Babkoff, 2013; Fostick et al., 2013). Listeners aged 61–82 years had thresholds for 15-ms tones that were 4.4 dB higher than listeners aged 21–40 years, even though the two groups had identical thresholds for 50-ms tones (Table 1 in Fostick and Babkoff, 2013).

Deafferentation, which comes with aging (Makary et al., 2011), could explain impaired short-tone detection in older listeners. One consequence of the stochastic nature of ANFs firing is that a loss of ANFs affects the representation of transient (short) waveforms more than sustained (long) waveforms. Thus, deafferentation may impair the detection of short sounds in a way perceptually similar to stochastic undersampling. Added to the finding of Lopez-Poveda and Barrios (2013) that stochastic undersampling leads to impaired intelligibility of speech in noise, the present results suggest that stochastic undersampling could be a common mechanism to explain how deafferentation results in impaired temporal processing and speech-in-noise intelligibility in older adults or in deafferented listeners with near normal audiometric thresholds. The present results also suggest that pure-tone audiometry for very short tones might be useful to assess the degree of deafferentation.

### Implications for Mechanisms of Sound Detectability

The present results are of interest for understanding the mechanisms underlying the detectability of sounds with different durations. As explained in the Introduction, the classical theories of “temporal integration”—based on a long-term integration of intensity—would predict detection thresholds unaltered by stochastic undersampling because undersampled and non-processed stimuli were equated for rms amplitude. Instead, undersampling short stimuli (with 1000 or 300 stochastic samplers) increased detection thresholds, and undersampling long stimuli (with 300 samplers) decreased detection thresholds. Thus, the present results are not consistent with the classical theories of “temporal integration”. The present results support instead the probabilistic theories of sound detectability (Meddis and Lecluyse, 2011; Heil et al., 2013a,b) that explain detection by a probability accumulation over time, with thresholds corresponding to a criterion number of detection events or to a single detection event.

**Figure 5:**
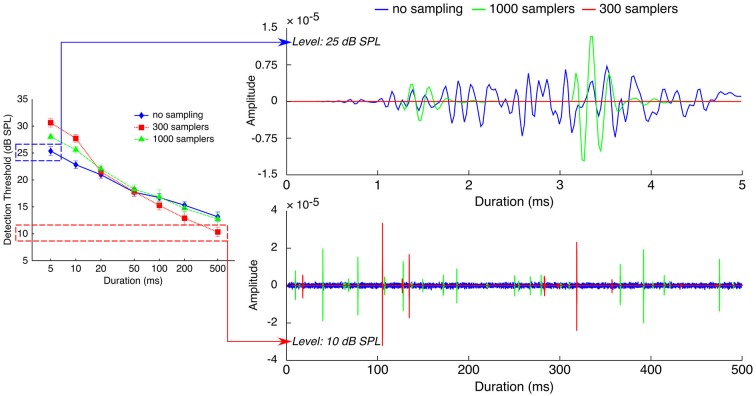
**Effects of stochastic undersampling on the waveforms of noise bursts with two different durations**. **Top**. 5-ms noises at 25 dB SPL, the measured threshold level for the no-sampling condition. **Bottom**. 500-ms noises at 10 dB SPL, the measured threshold level for the 300-samplers condition. See Section Implications for Mechanisms of Sound Detectability for a description.

Figure 5 illustrates how a probability accumulation of detection events could explain both the thresholds increase for short sounds and the thresholds decrease for long sounds. For short stimuli (5-ms stimuli on the top right panel), when plotting processed stimuli in the three sampling conditions and at the threshold level in the “no sampling” condition (25 dB SPL), it can be seen that processing with 1000 samplers kept very few samples, and that processing with 300 samplers removed all of the samples. If detectability depended on the occurrence of a criterion number of detection events, the limited number of samples in the two undersampled stimuli may not have been enough to trigger a criterion number of detection events. Thus, the limiting factor for detectability may have been the number of samples kept by the processing. Detectability in the two undersampled conditions was reached only at higher presentation levels, as higher intensities increase the probability of keeping any given sample, and hence increase the number of samples kept by the processing.

**Figure 6:**
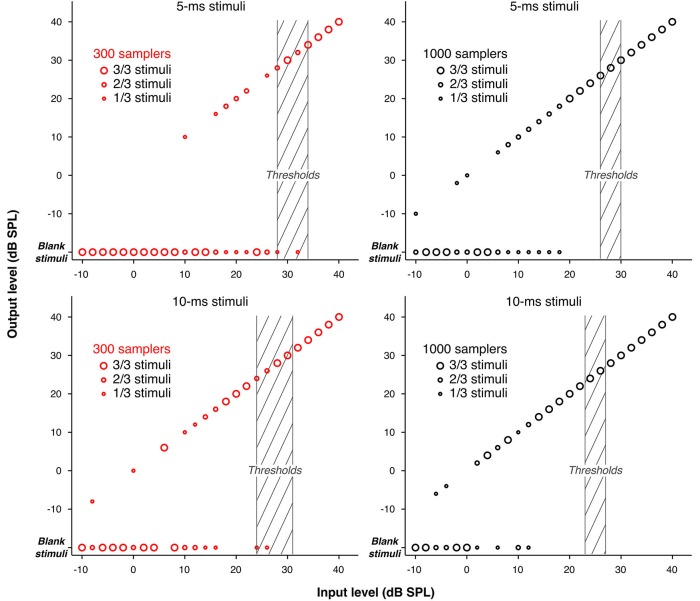
**Input/Output functions showing the actual levels of the pre-generated stimuli (“output level”) as a function of their level before stochastic undersampling (“input level”), for the two shortest durations (5 and 10 ms), processed with 300 and 1000 stochastic samplers**. Blank stimuli are also shown. The size of the circles marking the data points varies to indicate the proportions of blank and non-blank stimuli (either 1, 2, or 3 out of the 3 pre-generated stimuli) for each level. Levels above 40 dB SPL are not plotted because none of the stimuli pre-generated for those levels was blank. The shaded areas indicate the ranges of detection thresholds across participants.

As explained in Sections Stochastic Undersampling Vocoder and Stimuli (and illustrated in Figure 5), stimuli of low intensities and short durations were sometimes left blank by the stochastic undersampling process. This phenomenon was expected and is a consequence of the decreasing probability for samplers to generate “spikes” in response to lower intensities and shorter stimuli. Indeed, blank stimuli (effectively silence tokens) may be thought of as conditions were the original stimulus was too weak to elicit a neural response. The use of blank stimuli during the adaptive procedure may have contributed to elevating thresholds. Using only three pre-generated stimuli per condition may have exaggerated this effect. To clarify the contribution of blank stimuli to the elevation of thresholds, Figure 6 illustrates the actual levels (“output level”) of the pre-generated stimuli as a function of their level before stochastic undersampling (“input level”), for the two shortest durations processed with 300 and 1000 stochastic samplers. Blank stimuli are also plotted as a function of “input level”. The size of the symbols depicts the proportions of blank and non-blank stimuli, as indicated by the insets. Levels above 40 dB SPL were not plotted because none of the pre-generated stimuli was blank for those levels. It can be observed that blank stimuli were more frequent for lower intensities, for shorter durations, and when using 300 samplers. For the 300-samplers condition, blank stimuli were present within the range of individual threshold levels (shaded gray areas on Figure 6) and may have thus contributed to the observed elevation in threshold. On the other hand, for the 1000-samplers condition, blank stimuli were present only for levels about 10 dB or more below thresholds, hence their contribution to the thresholds elevation was probably negligible.

Contrary to short stimuli, detection of long (≥50 ms) stimuli was not limited by the number of samples since the easiest condition was the one with the fewest samples kept (i.e., thresholds were lowest in the 300-samplers condition). Instead, comparing on Figure 5 (bottom right panel) the waveforms of long stimuli in the three sampling conditions suggests that their peak amplitudes may have been the limiting factor. The bottom right panel of Figure 5 shows the waveforms of 500-ms stimuli in the three sampling conditions—all plotted at the threshold level of the 300-samplers condition (10 dB SPL). It can be seen that the 300-samplers condition was associated with larger peak amplitudes than the two other conditions. This is a result of the rms normalization: waveforms with fewer samples were scaled up to larger peak amplitudes to reach the rms amplitude of the control condition. Thus, a detection mechanism based on probability accumulation may have been more efficient in the 300-samplers condition because, at this low presentation level, only the larger peak amplitudes of the 300-samplers condition may have triggered detection events. In this view, the improvement of detection observed for long sounds would not be related to the stochastic undersampling *per se* but would be a side effect of the rms equalization.

### Limitations of the Present Model

The present results appear inconsistent with the findings of physiological studies on the effects of noise-induced deafferentation in rodents (Kujawa and Liberman, 2009; Lin et al., 2011; Furman et al., 2013). The present stochastic undersampling model predicts that the neural representation of low intensities will be more degraded than the neural representation of high intensities. In the aforementioned studies, by contrast, ABR thresholds recovered quickly after noise exposure while supra-threshold neural amplitudes were permanently reduced. ABR thresholds are independent from stimulus duration and are similar in magnitude to behavioral thresholds for short sounds (Gorga et al., 1984). Hence, the recovery of ABR thresholds after deafferentation appears inconsistent with the threshold elevation for short sounds reported here. This inconsistency, however, may be more apparent than real. Lin et al. (2011), p. 614, discussed that deafferentation likely elicited a small threshold elevation (<5 dB) that could not be seen because of the 5-dB step size used to measure ABR thresholds, because of the number of ears tested, and because of the variance in ABR amplitudes. Indeed, ABRs thresholds in Figure 1A of Furman et al. (2013) were slightly elevated (5–10 dB) at frequencies corresponding to the octave-band noise (4–8 kHz) used to cause deafferentation. The mean threshold elevations observed here for 5-ms stimuli were +2.6 dB when using 1000 samplers and +5.3 dB when using 300 samplers, relative to thresholds in the no sampling condition (Figure 2). Hence, the increase of thresholds for short sounds elicited by stochastic undersampling in the present study is not inconsistent with the aforementioned physiological studies.

Both the recovery of ABR thresholds and the reduction of neural amplitudes at supra-threshold intensities in the aforementioned physiological studies have been accounted for by a loss of low- and medium-SR fibers, which only discharge at medium and high intensities. This points to a limitation of the present study, namely that the vocoder used here and in Lopez-Poveda and Barrios (2013) does not simulate different types of fibers. The vocoder as currently implemented may thus be unable to simulate the shallower growth of ABR wave I with increasing level reported in previous studies (Kujawa and Liberman, 2009; Lin et al., 2011; Furman et al., 2013). This, however, is not a limitation of the stochastic undersampling principle *per se*. Future work will investigate whether implementing the three ANFs types in the vocoder changes the results of the present study.

Slightly elevated detection thresholds would be compatible with the aforementioned physiological studies if a fraction of the fibers deafferented in those studies were high-SR fibers. Figures given in Furman et al. (2013) suggest that this may have been the case. Furman et al. (2013) estimated that low- and medium-SR fibers represented 47% of the ANFs in control ears and 29% of the “surviving” ANFs in noise-exposed ears (p. 580). The total neural loss after noise exposure was roughly 40% (p. 583, hence “surviving” rate = ~60%). From these figures we can infer the following: (1) a control population of *M* fibers had 0.47 × *M* low- and medium-SR fibers; (2) after noise exposure, the total number of fibers lost was 0.40 × *M*; and (3) after noise exposure, the remaining number of low- and medium-SR fibers was 0.29 × (0.60 × *M*). Thus, the proportion of ANFs lost that were low- or medium-SR ANFs can be estimated as (0.47 × *M* − 0.29 × 0.60 × *M*)/(0.40 × *M*) = 0.74. This suggests that deafferentation can be associated with a comparatively less but still substantial loss of high-SR fibers (26%), and thus that threshold elevation can be caused by deafferentation if the degree of deafferentation is sufficient.

In that respect, it may be noted that the (arbitrarily chosen) number of samplers used in the present study might have simulated a greater amount of deafferentation than observed in noise-exposure studies (Kujawa and Liberman, 2009; Lin et al., 2011; Furman et al., 2013) and age-related deafferentation studies (Makary et al., 2011; Sergeyenko et al., 2013). The 300- and 1000-samplers conditions corresponded to 3000 and 10000 simulated ANFs when summed across the ten vocoding channels, which spanned 100–10000 Hz. The 100–10000-Hz frequency range can be estimated to cover 77% of the length of the basilar membrane (BM) using the almost-exponential frequency-position function of Greenwood (1990) (with parameters set so that the full BM length span 20–20000 Hz). Given that the density of inner hair cell (IHC) ribbon synapses is an inverted U-shaped function of the BM length peaking at 50% of the BM (Meyer et al., 2009), a BM section with a length of 77% the total BM length should encompass more than 77% of the total number of IHC ribbon synapses. Hence the 100–10000-Hz frequency range should correspond to at least 27000 ANFs in a non-deafferented ear (over a total of roughly 35000 ANFs; Miura et al., 2002), and the 300- and 1000-samplers conditions of the present study can be estimated to represent deafferentation rates of more than ~90% and 60% respectively. In noise-exposure studies, the deafferentation was estimated to be ~40% (Furman et al., 2013) and 50% (Kujawa and Liberman, 2009; Lin et al., 2011). Makary et al. (2011) reported a 30% loss of spiral ganglion cells in human temporal bones aged 91–100 years with no hair cells loss, and Sergeyenko et al. (2013) observed in mice that age-related cochlear synaptic degeneration (as indexed by presynaptic ribbons counts in IHC, their Figure 5C) was ~10% larger than the loss of spiral ganglion cells. Hence age-related deafferentation would appear to be capped at ~40%. Even considering that noise-related and age-related deafferentation would add up in real life, stochastic undersampling in the present study (and especially the 300-samplers condition) may have overestimated the amount of “ecological” age- and noise-related deafferentation.

### Stochastic Undersampling as a Mechanism for Age-Related Degradation of Temporal Processing

The present results support stochastic undersampling as a valid signal-processing analogy to explain the deteriorating effect of deafferentation on temporal processing. As deafferentation is associated with aging (Makary et al., 2011; Sergeyenko et al., 2013), and as aging is associated with temporal processing difficulties even in the absence of audiometric loss (CHABA, 1988; Fitzgibbons and Gordon-Salant, 2010; Lopez-Poveda, 2014), the present results also argue that stochastic undersampling could explain age-related temporal processing deficits.

Evidence for auditory deficits related to age *per se* is difficult to obtain in humans as older listeners most often have some degree of cochlear hearing loss that acts as a confounding variable (Tremblay and Burkard, 2007; Fitzgibbons and Gordon-Salant, 2010). Interestingly, steeper threshold/duration functions as observed in the present study are the opposite result to what is usually observed in patients with cochlear (mechanical) hearing loss. Patients with cochlear hearing loss usually show elevated detection thresholds for all sound durations, with the elevation being larger for longer durations, resulting in shallower threshold/duration functions than normal-hearing listeners (Florentine et al., 1988; Gerken et al., 1990; Plack and Skeels, 2007). The larger threshold increase for longer durations has been explained by an increase of the “absolute” sensory threshold, i.e., the minimum sound level below which not a single stochastic detection event is generated (Neubauer and Heil, 2004; Meddis and Lecluyse, 2011). The present results, together with the previous finding of elevated detection thresholds for short but not long sounds in older adults with age-corrected normal audiometric thresholds (Fostick and Babkoff, 2013; Fostick et al., 2013), suggest that brief tone audiometry could potentially be useful when trying to disentangle the effects of age *per se* from the effects of age-related cochlear hearing loss. One known limitation to the use of brief tone audiometry is the large variability between listeners (Olsen, 1987). Conflicting effects of age *per se* and of age-related cochlear hearing loss on the threshold/duration functions of older listeners may explain a part of this variability. Combining brief tone audiometry with measures of cochlear hearing loss—such as standard audiometry, audiometry in threshold-equalizing noise (Moore et al., 2000), distortion product otoacoustic emission (Dorn et al., 2001; Lopez-Poveda et al., 2009), or temporal-masking curves (Nelson et al., 2001; Lopez-Poveda and Johannesen, 2012)—might help isolate the “deafferentation component” of hearing deficits in older listeners.

It should be stressed that the stochastic undersampling analogy was not conceived as a model of the physiological response of deafferented auditory nerves. Instead, it was meant to simulate a reduction of information in the nerve on the basis of the stochastic firing properties of neurons. However, stochastic undersampling in the nerve is not the only possible explanation for impaired temporal processing. Zeng et al. (2005) argued that the degraded temporal processing of auditory neuropathy patients could be explained by reduced synchronization between ANF responses or by deafferentation. Pichora-Fuller et al. (2007) found that simulating desynchronization by jittering the frequency components of speech stimuli could explain the poorer speech-in-noise intelligibility of older listeners with normal audiometric thresholds. Stochastic undersampling and deafferentation, however, offer a more parsimonious explanation than desynchronization because they do not postulate changes in the temporal properties of individual ANFs. In other words, according to the stochastic undersampling view, older adults may not have a “slower-than-normal” auditory processing but, more simply, they would have fewer functional ANFs.

A loss of functional ANFs appears early in the aging process as a consequence of cochlear synaptopathy (Sergeyenko et al., 2013). Age-related alterations in the auditory system cannot, however, be reduced to deafferentation. For example, age-related auditory deficits have been associated with alterations of the cochlear lateral wall that lead to a reduction of the endocochlear potential (Schmiedt, 2010) and hence to IHC and outer hair cell (OHC) dysfunction (Meddis et al., 2013; Saremi and Stenfelt, 2013). Stochastic undersampling may be used to simulate this and other pathologies. Indeed, stochastic undersampling can occur either by reducing the number of samplers (deafferentation) or by reducing the individual probability of firing of (sub)populations of samplers (ANFs) (Lopez-Poveda, 2014). Any alteration of the ear that would result in reduced probabilities of neural firing would also result in some samples of the stimulus waveform not being encoded in the auditory nerve, hence in a form of stochastic undersampling not related to deafferentation. Future work with vocoder implementations that include “abnormal” firing probabilities for the three types of ANFs may provide a way to study the effects of various age-related auditory alterations independently. For example, OHC dysfunction would reduce auditory sensitivity to soft sounds and might be studied by reducing the probability of firing of high-spontaneous rate fibers, whereas deafferentation should be implemented as a reduction in the number of fibers, particularly of those with low- and medium-spontaneous rates. Age-related auditory deficits have also been associated with alterations central to the auditory nerve. For example, aging comes with a progressive weakening of GABAergic systems (i.e., reduced inhibitory neurotransmission) throughout the central auditory system (Canlon et al., 2010), which may at least partly result from a progressive deafferentation (Caspary et al., 1995). Deafferentation central to the auditory nerve has also been suggested by ABR data showing an age-related amplitude reduction of wave III apparent even after controlling for cochlear and auditory nerve changes reflected in wave I amplitude (Konrad-Martin et al., 2012). The stochastic undersampling analogy may thus be used also to study age-related neural alterations central to the auditory nerve, by using probability-intensity functions characteristic of central neurons instead of functions characteristic of ANFs.

## Conclusions

1. Stochastic undersampling impairs the detection of short (<20 ms) sounds, consistently with the impairments observed in auditory neuropathy patients and in older listeners with (near) normal audiometric thresholds.
2. Insofar as deafferentation can produce stochastic undersampling and deafferentation comes with aging, the present results suggest that some of the temporal processing deficits of older adults could be due to deafferentation.
3. The present results suggest that deafferentation might be diagnosed using pure-tone audiometry with short tones.
4. Stochastic undersampling, as implemented here, impaired the detection of short sounds and, in certain conditions, improved the detection of long sounds. As rms amplitudes were equalized across undersampling conditions, the present results are not consistent with theories of detectability based on the integration of energy over the stimulus duration (the seminal temporal integration theory). Instead, the present results support probabilistic theories of detectability.

## Conflict of Interest Statement

The authors declare that the research was conducted in the absence of any commercial or financial relationships that could be construed as a potential conflict of interest.
